# Comprehensive Profiling of Gene Copy Number Alterations Predicts Patient Prognosis in Resected Stages I–III Lung Adenocarcinoma

**DOI:** 10.3389/fonc.2019.00556

**Published:** 2019-08-06

**Authors:** Xiaohong Han, Qiaoyun Tan, Sheng Yang, Junling Li, Jianping Xu, Xuezhi Hao, Xingsheng Hu, Puyuan Xing, Yutao Liu, Lin Lin, Lin Gui, Yan Qin, Jianliang Yang, Peng Liu, Xingyuan Wang, Wumin Dai, Dongmei Lin, Hua Lin, Yuankai Shi

**Affiliations:** ^1^Department of Medical Oncology, National Cancer Center/National Clinical Research Center for Cancer/Cancer Hospital, Chinese Academy of Medical Sciences & Peking Union Medical College, Beijing Key Laboratory of Clinical Study on Anticancer Molecular Targeted Drugs, Beijing, China; ^2^Department of Clinical Laboratory, National Cancer Center/National Clinical Research Center for Cancer/Cancer Hospital, Chinese Academy of Medical Sciences & Peking Union Medical College, Beijing, China; ^3^Department of Pathology, National Cancer Center/National Clinical Research Center for Cancer/Cancer Hospital, Chinese Academy of Medical Sciences & Peking Union Medical College, Beijing, China; ^4^Department of Medical Record, National Cancer Center/National Clinical Research Center for Cancer/Cancer Hospital, Chinese Academy of Medical Sciences & Peking Union Medical College, Beijing, China

**Keywords:** lung adenocarcinoma, copy number alteration, prognosis, predict, adjuvant chemotherapy

## Abstract

**Background:** Lung adenocarcinoma (LUAD) possesses a poor prognosis with a low 5-year survival rate even for stages I-III resected patients, it is thus critical to understand the determinants that affect the survival and discover new potentially prognostic biomarkers. Somatic copy number alterations (CNAs) are major source of genomic variations driving tumor evolution, CNAs screening may identify prognostic biomarkers.

**Methods:** Oncoscan MIP array was used to analyze the patterns of CNAs on formalin fixed paraffin embedded(FFPE) tumor specimens from 163 consecutive stage I-III resected LUAD patients, 145 out of which received platinum-based adjuvant chemotherapy.

**Results:** Of the 163 patients, 91(55.8%) were recurred within 3 years after surgery. The most common aberrations in our cohort were 1q, 5p, 5q, 7p, 8q, 14p, 16p, 17q, 20q for copy number gains and 8p, 9p, 13p, 16q, 18q for losses. The GISTIC2 analysis produced 45 amplification peaks and 40 deletion peaks, involving some reported genes *TERT, EGFR, MYC, CCND1, CDK4, MDM2, ERBB2, NKX2-1, CCNE1*, and *CDKN2A*, most of which were consistent with TCGA database. The amplifications of 12p12.1 (*CMAS, GOLT1B, YS2, LDHB, RECQL, ETNK1, IAPP, PYROXD1, KRAS*) and *KDM5A* were correlated with worse prognosis in our cohort, this result was further validated in 506 LUAD patients from TCGA. In addition, 163 patients could be well-classified into five groups, and the clinical outcomes were significantly different based on threshold copy number at reoccurring alteration peaks. Among the 145 patients who received adjuvant chemotherapy, focal amplification of *ERBB2* and deletion of 4q34.3 were found to be specific in relapsed patients, this result was validated in an independent group of Imielinski et al., demonstrating these two CNAs may contribute to resected LUAD recurrence after adjuvant chemotherapy.

**Conclusion:** This study suggests that CNAs profiling may be a potential prognostic classifier in resected LAUD patients. Amplifications of 12p12.1 and *KDM5A* might be prognostic biomarkers for LUAD, and amplification of *ERBB2* and deletion of 4q34.3 predicted early relapse after adjuvant chemotherapy. These novel findings may provide implication for better implementation of precision therapy for lung cancer patients.

## Introduction

About 85 % of lung cancers are non-small cell lung cancer (NSCLC), the majority of which are lung adenocarcinoma (LUAD) and squamous cell carcinoma subtypes (1). Patients diagnosed with NSCLC usually present with advanced stages and experience a dismal survival rate. Even for patients with surgically resectable stage I to III diseases, relapse is unfortunately common (2). Currently, the clinical principle prognosis prediction for NSCLC is tumor extension, characterized by tumor node metastasis (TNM) staging (3). However, the clinical outcome varies among individuals sharing the same clinical features, suggesting the existence of unknown factors that influence the disease outcomes, this necessitates the discovery of new potentially prognostic biomarkers which can identify patients with higher risk of relapse after surgical resection.

As a major source of genomic variations driving tumor evolution, somatic copy number alterations (CNAs) may provide potential prognostic information. Several studies have addressed the prognosis value of CNA patterns in various cancers. Yu et al. evaluated CNAs in 81 esophageal squamous cell cancer patients, and found four genes were significantly associated with the patients‘ prognosis (4). In breast cancer, gene CNA signature patterns were proven to be correlated with clinical features and survival (5). In NSCLC, a genotype classifier based on CNA differences between LUAD and squamous cell carcinoma has been reported and gained our understanding of carcinogenesis of lung cancer (6, 7). As a fact that LUAD composed of the majority of lung cancer, and the CNA spectrum difference do exist between LUAD and squamous cell carcinoma, it is thus important to explore the prognostic predictive value of CNA in LUAD subtype. On the other hand, most of the samples in clinical settings are formalin-fixed and paraffin-embedded (FFPE), and DNA-based tests are more robust when applied to FFPE tissues, thus, identifying prognostic markers based on CNA from FFPE samples may be of potential clinical significance.

The goal of this study was to evaluate the genetic CNA profiles in LUAD and to assess the predictive value of CNA for survival benefits, subsequently we investigated potential CNA associated with adjuvant chemotherapy in a group of patients who received platinum-based adjuvant chemotherapy treatment. To this end, we extensively analyzed the CNA data obtained by Oncoscan MIP array using FFPE samples from 163 LUAD patients and correlated these results with the clinical survival outcomes.

## Materials and Methods

### Sample Collection and DNA Extraction

A total of 190 patients were consecutively recruited in National Cancer Center/National Clinical Research Center for Cancer/Cancer Hospital, Chinese Academy of Medical Sciences & Peking Union Medical College between 2010 and 2014. All patients were pathologically confirmed with LUAD and treated with operation, clinical information was obtained from hospital electronic medical database. This was a retrospective study in nature, the study was conducted in accordance with the ethical guidelines of the National Cancer Center/National Clinical Research Center for Cancer/Cancer Hospital, Peking Union Medical College and Chinese Academy of Medical Sciences (NCC2017GKZ-01), and BGI Genomics (BGI-IRB18004). Tumor specimens were collected and paraffin blocks was prepared following standard process at the Department of Pathology, about 3–5 slices of 10 μm of thickness were collected from the paraffin block and made into an H&E-stained slide for DNA extraction. The tumoral area was manually dissected with scalpel following identification by superimposition of a H&E stained glass, and digested at 56°C in ATL buffer over-night with proteinase K (Qiagen) in. DNA extraction was then continually performed with QIAamp DNA micro kit (Qiagen) following manufacturer's instructions. Spectrophotometric (Nanodrop) were used to quantify DNA concentration.

### Copy Number Analysis

Genome-wide estimation of CNAs was performed using the OncoScan FFPE Assay Kit (8) following the manufacturer's instructions. Briefly, 80 ng of FFPE DNA at a concentration of 6.6 mL at 12 ng/mL was used for the analysis. The DNA was incubated with biotin-labeled molecular inversion probes 16 h at 58°C. Subsequently, the circularized DNA is subjected to endonuclease cleavage, which generates fragments with common regions on the 30 and 50 ends that are subsequently used to amplify the fragments. Post amplification, the fragments are subjected to HaeIII endonuclease digestion and hybridized to the array overnight, stained, and scanned. Scanning results from both arrays are combined by the OncoScan Console software version 1.2 (Affymetrix) and subjected to further analysis and visualization by ASCAT (9) as implemented in the Nexus Express software. As per the manufacturer's recommendation, a gene copy number of one or less was considered as a copy number loss, copy number of three as gain, and four or more as high gain. Significantly focal copy number gains or losses were evaluated with GISTIC 2.0 (10) using default parameters.

### NMF Analysis

Non-negative matrix factorization (NMF) (11) is a dimensionality reduction method which we can factorize a high-dimensional matrix profile to several matrices, thus, making the resulting matrices easier to inspect. Results from NMF vary slightly based on initial conditions. Here, we used different initial conditions with *k* = 2–10 for thresholded copy number profile of focal alteration peaks, Hence, we obtain a consensus plot (and cophenetic coefficient) for each *k* = 2 to 10 and seek values of *k* for which *k* is close to 1. Our data provided compelling evidence in support of *k* = 4 clusters (Supplementary Figure 4), suggesting that there was evidence for four consensus clusters. This result was also observed in two validation datasets.

### Validation Datasets

Validation datasets included 159 LUADs from the cohort of Imielinski et al. (12), and 506 LUADs from TCGA of which whole genome CNAs (Affymetrix SNP 6.0 SNP array) and mRNA expressions (RNA-seq V2 RSEM) data were downloaded from TCGA portal.

### Statistical Analysis

Statistical analysis was performed using R software (version R 3.2.3). The Student's *t*-test was used to compare the significant differences between two groups. All statistical tests were two-sided. The Fisher's exact test was used to determine association between two categories. Statistical significance was declared if the *P*-value was <0.05. Survival time distribution in the survival analysis was assessed by the Kaplan–Meier method using log-rank tests. We constructed a multivariate model to compute the HR based on the CNA status of select gene, including gender (male vs. female), age at surgery (≥58 vs. <58 years), family history (yes vs. no), smoking history (yes vs. no), high blood pressure (yes vs. no), diabetes (yes vs. no), tumor location (right vs. left), TNM stage (III vs. I and II), blood vessel invasion (yes vs. no), pleura invasion (yes vs. no), lymphnode metastasis (yes vs. no), and chemotherapy (yes vs. no). Interactions were assessed by including the cross-product of the CNA status and another variable of interest in a Cox model.

## Results

### Patient Clinical Characteristics

**Figure 1:**
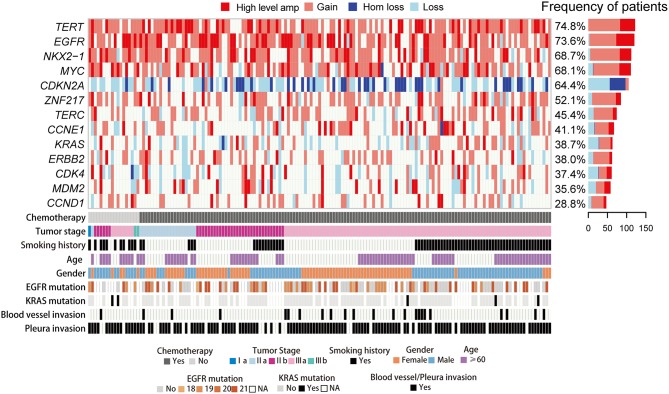
CNA spectrum and clinical characteristics of 163 LUAD cases. Heat map shows somatic CNAs with different status. Bars in the right represent the proportion of the CNAs identified in LUAD. The panel in the bottom shows key clinicopathological characteristics.

Of 190 LUAD patients consecutively recruited between 2010 and 2014, 27 failed the CNA genotyping might because of degradation and insufficient quantity of DNA obtained from FFPE samples, leaving 163 patients eligible for CNA and survival analyses. The clinical features of these 163 patients are shown in Supplementary Table 1 and Figure 1. The proportion of male (53%) and female (47%) were similar, and more than half (53%) were non-smokers. The median age in diagnosis was 58 (25–78 years) and the median follow-up was 5.62 years (0.58–7.19 years). The cohort included 1 stage I, 104 stage II, 58 stage III cases. All 163 patients received operation and 145 (88.9%) out of which received adjuvant chemotherapy treatment. All tumors were chemotherapy-naïve, primary resection specimens. Sample acquisition and processing details are provided in the Experimental Procedures.

### CNA Detection and Validation

**Figure 2:**
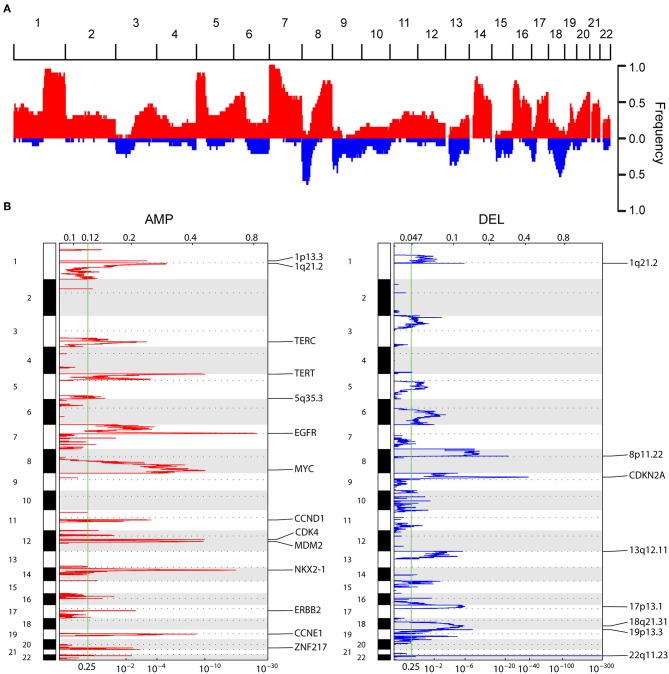
Composite copy number profiles for LUAD tumors. **(A)** Frequency plot s of CNAs distribution in 163 LUAD patients, red and blue indicate gain and loss, respectively **(B)** GISTIC2.0 focal amplifications (red) and deletions (blue) for 163 LUADs. Peaks with an FDR < 0.25 are annotated with candidate oncogenes, tumor suppressors or cytobands.

Eighty nanogram FFPE gDNA has been extracted from 163 FFPE samples and purified as input to the OncoScan CNA Plus Assay to explore the whole genome CNAs and capture the alleles of nearly 220,000 SNPs at carefully selected genomic locations. Microarray data was transformed into genome-wide segmentation and allele imbalance data by using ASCAT (9). Our results showed the very similar landscape of somatic CNAs with TCGA cohort. The most frequent arm-level aberrations identified were 1q, 5p, 5q, 7p, 8q, 14p, 16p, 17q, 20q for copy number gains and 8p, 9p, 13p, 16q, 18q for losses (Figure 2A). GISTIC2 analysis generated 45 recurrent amplification peaks and 40 recurrent deletion peaks including some previously reported genes *TERT, EGFR, MYC, CCND1, CDK4, MDM2, ERBB2, NKX2-1, CCNE1*, and *CDKN2A* (Figure 2B), most of which were consistent with previous results in TCGA analyses (13). We next investigated CNAs of these genes across 163 LUADs, and found copy number gain was prominent in *TERT* (74.8%), *EGFR* (73.6%), *NKX2-1* (68.7%), *MYC* (68.1%), whereas loss was predominant in *CDKN2A* (64.4%) (Figures 1, 2B).

### Association Analysis With Prognosis

The multivariate proportional hazards analysis was applied to clinical variables, TNM stage and chemotherapy were found to be independent prognostic factors in our data (Supplementary Figure 1). Compared with patients received only operation, adjuvant chemotherapy significantly improved the overall survival (OS) (*p* < 0.001). This significance which remained in the multiple analysis confirmed the conclusion as a result of several large clinical trials. There were no additional parameters other than tumor stage robustly associated with OS in the multivariate analysis (including all factors with *p*-values <0.05 from the univariate analysis). Stage III patients had a 1.82-fold increased risk of death compared with stage I and II LUAD patients.

**Figure 3:**
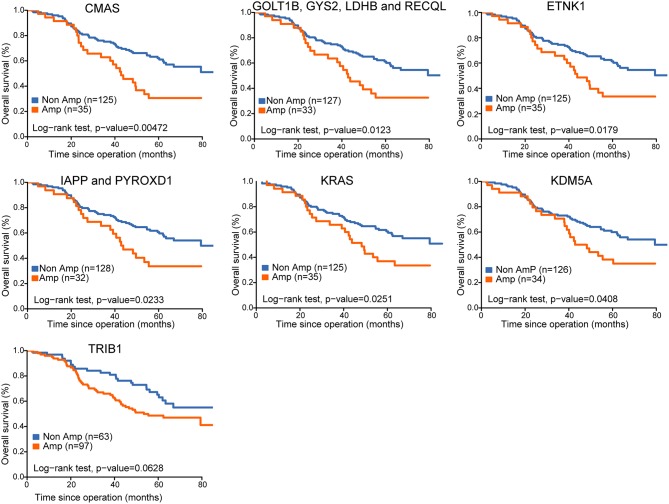
Kaplan-Meier survival curves for survival of lung adenocarcinoma with CNA of select genes.

To explore the potential prognostic role of CNAs changes, we applied the univariate proportional hazards analysis to CNAs of all genes, and discovered 16 significantly amplified CNAs that derived increased hazards of death. We further determined whether the association of these CNAs on cancer-specific survival was modified by any of the clinical pathologic variables evaluated. Among 16 genes of interest, 11 genes from 2 locations (12p12.1 and 12p13.33) remained associated with OS in multiple analyses (Table 1). Kaplan–Meier analysis indicated amplifications of 12p12.1 (*CMAS, GOLT1B, GYS2, LDHB, RECQL, ETNK1, IAPP, PYROXD1*, and *KRAS*) and 12p13.33 (*KDM5A*) were associated with worse prognosis in our cohort (Figure 3).

**Table 1:** Cox regression analyses for CNAs associated with survival.

**Characteristics (Amp vs. Non-Amp)**	**Univariate analysis**		**Multivariate analysis**	
	**HR (95% CI)**	***P***	**HR (95% CI)**	***P***
CMAS	1.99 (1.22~3.23)	0.006	2.80 (1.60~4.88)	<0.001
GOLT1B,GYS2,LDHB,RECQL	1.87 (1.14~3.07)	0.014	2.48 (1.41~4.37)	0.002
ETNK1	1.79 (1.10~2.93)	0.020	2.26 (1.60~3.94)	0.004
IAPP,PYROXD1	1.78 (1.07~2.94)	0.025	2.30 (1.30~4.07)	0.004
KRAS	1.74 (1.06~2.84)	0.027	2.14 (1.24~3.71)	0.006
KDM5A	1.67 (1.02~2.74)	0.043	1.88 (1.08~3.26)	0.026
TRIB1	1.57 (0.97~2.54)	0.065	1.62 (0.982~2.68)	0.059

Whole genome CNAs and gene expression data of 506 lung adenocarcinoma were downloaded from TCGA database, as shown in Supplementary Figure 2. In comparison to the unamplified genes, amplifications of 12p12.1 (*CMAS, GOLT1B, GYS2, IAPP, KRAS, LDHB, PYROXD1, RECQL*, and *ETNK1*) predicted poorer OS (*p* < 0.05), and the expression of these genes increased significantly accompanying the increased copy number (Supplementary Figure 3). There was a trend toward an OS benefit from *TRIB1* non-amplification vs. amplification group in our cohort (*p* = 0.062), which was further validated in TCGA with a marginally significant association (*p* = 0.049). The prognostic impact of *KDM5A* amplification was seen on both univariable and multivariable analysis, with a significant difference in gene expression (*p* < 0.001). Of note, this is the first time to find *KDM5A* CNA was significantly associated with prognosis in resected LUAD.

### Classification of LUAD Based on CNA

**Figure 4:**
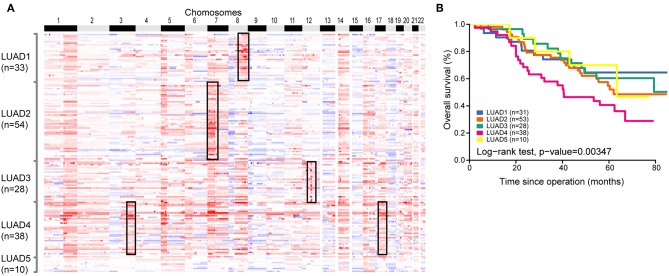
Copy number subtypes of 163 LUAD genomes. **(A)** In the heat map, SCNAs of each tumor which was assigned to five subgroups (vertical axis) are plotted by chromosomal position (horizontal axis). **(B)** The Kaplan-Meier plot depicting overall survival (OS) within 163 LUADs stratified by the classification.

To explore the intratumor heterogeneity of LUAD, we performed molecular classification analysis by using NMF to cluster copy number profiles of 163 LUAD tumors. These tumors were clustered based on thresholded copy number of focal alteration peaks which was identified by GISTIC 2.0 analysis. This analysis defined four clusters with highest cophenetic coefficient (Supplementary Figure 4). We found that these clusters exhibit exclusive focal amplification of well-known oncogenes in lung cancer, finally, the 163 lung adenocarcinomas were reliably divided into five groups: LUAD1 (*n* = 33, 20.2%), LUAD2 (*n* = 54, 33.1%), LUAD3 (*n* = 28, 17.2%), LUAD4 (*n* = 38, 23.3%), LUAD5 (*n* = 10, 6.2%) (Figure 4A). The first group of tumors was significantly enriched for amplification of 8q on which a driver gene *MYC* is located. *MYC* is associated with genomic instability by promoting cell cycle progression and chromosome aneuploidy (14). The second group was enriched for a widely used clinical target gene *EGFR* (LUAD2). Focal amplification of *MDM2* which play a role in tumorigenesis through inhibition of P53 protein was concentrated in LUAD3 group. The remaining group 4 was enriched for overexpression of *TERC* and *ERBB2 (HER2), ERBB2* is a member of the human epidermal growth factor receptor family involved in receptor tyrosine kinases which regulate the modulation of cell proliferation, apoptosis, and cell mortality (15). Subsequently, we manually ascertained several tumors without any amplification of genes mentioned above, and assigned them a new group (LUAD5). The classifier was also validated in two additional independent sets [Imielinski et al. (12) and TCGA (13)], all subtypes were identified with comparable proportions of samples (Supplementary Figures 4, 5).

Evaluation of the clinical characteristics of these subtypes suggested no significant correlation between them. Moreover, Kaplan-Meier survival analysis revealed significant differences in OS of the five subgroups and confirmed a poor prognosis for patients with LUAD4 subgroup (Figure 4B). Similar prognosis result was also observed in validation dataset (Supplementary Figure 5). Cumulatively, our classification system might provide useful information for risk stratification and treatment.

### ERBB2 Amplification Associated With Relapse After Adjuvant Chemotherapy

**Figure 5:**
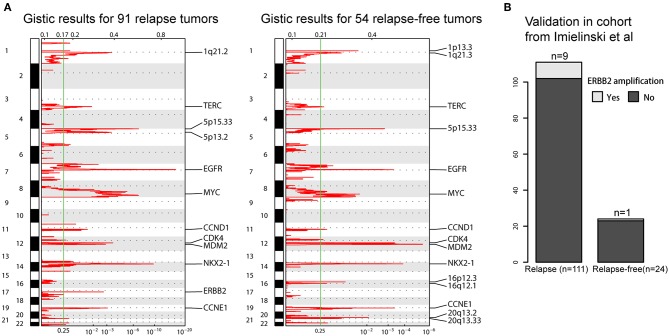
Focal copy number peaks specified for post-operative relapse LUADs. **(A)** GISTIC2.0 focal amplifications (red) for post-operative relapse and relapse-free LUADs. Peaks with an FDR < 0.25 are annotated with candidate oncogenes or cytobands. **(B)** Barplot shows the comparison on the ERBB2 amplification in validation sets.

The prognosis of LUAD has improved due to the standardization of adjuvant chemotherapy and the adoption of target therapy, however, the survival states of patients with recurrence after surgical resection are not well-studied. We speculated that CNA may become molecular biomarkers for helping inform clinical therapeutic strategies for post-surgical recurrence. Here, we included 145 patients who have received adjuvant chemotherapy and separated them into two groups based on whether LUAD was recurred within 3 years (91 relapse and 54 relapse-free). GISTIC 2.0 analyses were applied to these two groups to identify mutually exclusive foal amp/del makers. We saw a recurrent amplification at 17q12 at the locus containing *ERBB2* only in relapse tumors, which was further validated in cohort of Imielinski et al. (Figure 5). Amplification or over expression of this gene has occurred in ~2–5% of LUAD (16). They have been identified as oncogenic drivers and associated with a poor prognosis in lung cancer (17). Previous studies have also suggested that ERBB2 overexpression is strongly associated with increased chemotherapy resistance leading to increase disease recurrence in breast cancers (18). Our result indicated that ERBB2 amplification may also have a role in regulating recurrence in LUAD. There were many other specific CNAs, such as deletion of 4q34.3 for relapse tumors, amplifications of 16p12.3 and 16q12.1 and deletion of 21q22.3 for relapse-free tumors (Supplementary Figures 6, 7). More detailed integrated genomic and transcriptomic sequencing analyses will be needed to elaborate the underlying mechanisms in the future.

## Discussion

In this study, we sought to explore the CNA spectrum of lung adenocarcinoma patients and determine whether the CNA profiling could provide more insight into the different prognosis in patients receiving adjuvant chemotherapy.

Here, we tested the CNA in 163 LUAD patients. Our results show the greatest defections involved gains in 1q, 5p, 5q, 7p, 8q, 14p, 16p, 17q, 20q and losses in 8p, 9p, 13p, 16q, 18q, which shared similar CNAs landscape with TCGA database. Consistent with previous data, LUAD frequently displays gains in driver genes (19), and we identified frequent amplifications in known genes: *TERT, EGFR, MYC, CCND1, CDK4, MDM2, ERBB2, NKX2-1, CCNE1*, and this was further validated in TCGA database. In line with previous report (6), telomerase reverse transcriptase (*TERT*), located in 5p15, is the most frequently amplified gene in our result. Gains of oncogenes including *EGFR* (7p12), *ERBB2* (17q12), *KRAS* in the EGFR pathway were characteristic for LUAD and contribute to cell proliferation and tumor development, suggesting this is a core requirement for LUAD pathogenesis. *NKX2-1* was firstly known to contribute to normal lung cells differentiation (20, 21), later it was found to be a specific tissue marker in LUAD as patients carry increased *NKX2-1* gene amplification and protein expression compared to normal cells (22), which was not identified in other cancers types of breast, prostate, colon and pancreas (23–25), indicating *NKX2-1* was an oncogene specific for lung cancer, and now it's commonly used for differential diagnosis in clinical practice (26). However, the oncogene role and function mechanism remain incompletely clarified. Recently, Deborah et al. reported that *NKX2-1* targeted on Selenium binding protein 1 (Selenbp1) led to LUAD growth inhibition (27). In our study, we showed that *NKX2-1* amplification is commonly occurred in LUAD, which permitted the possibility of further function investigation for LUAD. Apart from gene gains, *CDKN2A*, which is frequently mutated or deleted in a wide variety of tumors, was significantly deleted in our cohort. *CDKN2A* is known to be a tumor suppressor gene (28, 29). For malignant mesothelioma, *CDKN2A* deletion was reported to be potential diagnostic and prognostic marker (30). Though different mechanisms of *CDKN2A* deletion exist in various human cancers (31), the *CDKN2A* tumor-suppression functions can be attenuated through point mutation, promoter hypermethylation, or deletion, and this could be one reason why this locus bears a high frequency of homozygous deletion.

When we analyzed the association of CNAs with long time survival, our analysis revealed 11 genes from two locations associated with worse prognosis, including amplifications of 12p12.1 (*CMAS, GOLT1B, YS2, LDHB, RECQL, ETNK1, IAPP, PYROXD1, KRAS*) and 12p13.33 (*KDM5A*). Former studies detected amplification of 12p12.1 in testicular germ cell tumors (32, 33). In lung adenocarcinoma, 18 genes (*SLCO1A2, PYROXD1, RECQL, LDHB, CMAS, KIAA0528, ETNK1, ASUN, FGFR1OP2, TM7SF3, MED21, MRPS35, KLHDC5, CCDC91, FGD4, DNM1L, YASR2*, and *KRAS*) in 12p12.1 were found to have increased copy number and coamplified with *KRAS*, 88.9% (8/9) genes in our study were included. Among them, *LDHB* amplification was found to be associated with RAS pathway activation. The elevated LDHB level in serum was validated to correlate with clinical stage and poor outcome of lung cancer (34, 35), indicating its potential to predict prognosis in lung cancer patients. *CMAS* is a protein-coding gene and participates in protein metabolism, it was reported to significantly associated with metastasis which decreases breast cancer survival (36). *KDM5A* was identified as a histone demethylase which represses transcription of its targeted genes (37, 38). It was reported that *KDM5A* is required to develop a metastable chromatin state that enables drug resistance in breast cancer (39) and lung cancer treated with EGFR-TKI (40). As far as we know, this is the first time to find *KDM5A* correlated with clinical outcome in LUAD patients receiving adjuvant chemotherapy in our study. Together, the critical role of *KDM5A* played in chemotherapy and targeted therapy drug tolerance yield an opportunity to develop pharmacologic inhibitors of *KDM5A* which potentially prevent drug resistance and improve treatment efficacy.

Moreover, we have built a molecular classifier for LUAD based on CNA profile, and identified five distinct subtypes (LUAD1, LUAD2, LUAD3, LUAD4, and LUAD5). These five distinct subtypes were validated in independent primary data sets. Our findings revealed that common CNA alterations are helpful for subtype classification. Each subgroup CNA with previously published focal amplifications helped improving the biological relevance of the stratification. Indeed, this analysis suggested that different types of LUAD may exhibit distinct focal amplifications, including *MYC, ERBB2, EGFR*, and *MDM2*. The *ERBB2*-amplificated subtype was well-identified with poor prognosis. This LUAD4 subtype is also observed with amplification of *TERC*, a gene located at chromosome 3 which serves as a template for the telomere repeat, its overexpression may be involved in oncogenesis (41). The further detection and investigation of the LUAD4 tumors will potentially give rise to optimized therapy for LUAD patients. In addition, there was no significant relationship between our classifier and clinical pathological stage, indicating that tumor subgroup might be established at the initial stages.

In the present study, we also attempted to explore the association between copy number profile and post-operative chemotherapy recurrence. *ERBB2* amplification and 4q34.3 deletion were specially identified in LUAD patients who had tumor recurrence occurring within 3 years after therapy. Previous studies are mainly concentrated on ERBB2-mutated metastatic NSCLC (42), to our knowledge, our study is the first to show *ERBB2* amplification is associated with the resistance of adjuvant chemtherapy and recurrence in LUAD patients, monoclonal antibody trastuzumab and pertuzumab which target ERBB2 may help guide therapeutic strategies for post-operative relapse for LUAD. This result was also validated in an independent data set. Collectively, our findings suggested that *ERBB2* amplification plays a key role in mediating resistance to chemotherapy in LUAD, and further studies involving large LUAD cohorts with completely clinical data and other omics, may facilitate guiding therapeutic strategies.

In conclusion, we have shown that CNAs might be a potential prognostic classifier in resected LUAD patients. In addition, we found that amplifications of 12p12.1 and *KDM5A* were associated with worse clinical outcome for resected LUAD, and amplification of *ERBB2* and deletion of 4q34.3 were associated with adjuvant chemotherapy resistance. These novel findings support a prognostic potential of CNAs and may provide certain implication for better clinical decision.

## Data Availability

All data included in this study are available upon request by contact with the corresponding author.

## Ethics Statement

This was a retrospective study in nature, and was conducted in accordance with the Declaration of Helsinki and all participants were above the age of 18. The study was approved by the Independent Review Board (IRB) of Cancer Hospital, Chinese Academy of Medical Sciences and BGI Genomics. The data will be available on request.

## Author Contributions

YS and XHan contributed to the concept and design of this study. YS, XHan, QT, SY, JL, JX, XHao, XHu, PX, YL, LL, LG, YQ, JY, PL, XW, WD, DL, and HL performed acquisition of data (collected samples of patients, provided patients clinical information etc.). QT, XHan, and YS wrote, reviewed and revised the manuscript. QT, SY, and HL performed administrative, technical, or material support (i.e., reporting or organizing data, constructing databases). YS and XHan supervised the study.

### Conflict of Interest Statement

The authors declare that the research was conducted in the absence of any commercial or financial relationships that could be construed as a potential conflict of interest.

## Supplementary Material

Supplementary Figure 1 Multivariate analysis of clinical information with prognosis. **(A)** The panel shows hazard ratios (HR) of clinicopathological characteristics for cancer-specific survival. **(B)** The Kaplan-Meier graphs depicting overall survival (OS) associated with chemotherapy and TNM stage.

Supplementary Figure 2 Kaplan-Meier survival curves for survival of lung adenocarcinoma with CNA of select genes in TCGA dataset.

Supplementary Figure 3 Analyses of select genes expression levels by corresponding CNA status in 506 LUAD patients' tumors from the TCGA Project.

Supplementary Figure 4 NMF consensus clustering analysis and cophenetic coefficient for cluster *k* = 2 to *k* = 10. **(A)** our cohort. **(B)** The cohort from Imielinski et al. **(C)** TCGA cohort.

Supplementary Figure 5 Classification of validation datasets. **(A)** The cohort from Imielinski et al. **(B)** TCGA cohort.

Supplementary Figure 6 GISTIC2.0 focal deletions (red) for post-operative relapse and relapse-free LUADs. Peaks with an FDR < 0.25 are annotated with candidate tumor suppressors or cytobands.

Supplementary Figure 7 Barplot shows the comparison on the LOC285501 deletion in validation sets.

Supplementary Table 1 Patient characteristics of 163 patients.
