# Supplementary figures and images for: Comprehensive Profiling of Gene Copy Number Alterations Predicts Patient Prognosis in Resected Stages I–III Lung Adenocarcinoma
Source: Front Oncol. 2019 Aug 6;9:556. doi: 10.3389/fonc.2019.00556 (PMC6691340; doi:10.3389/fonc.2019.00556)

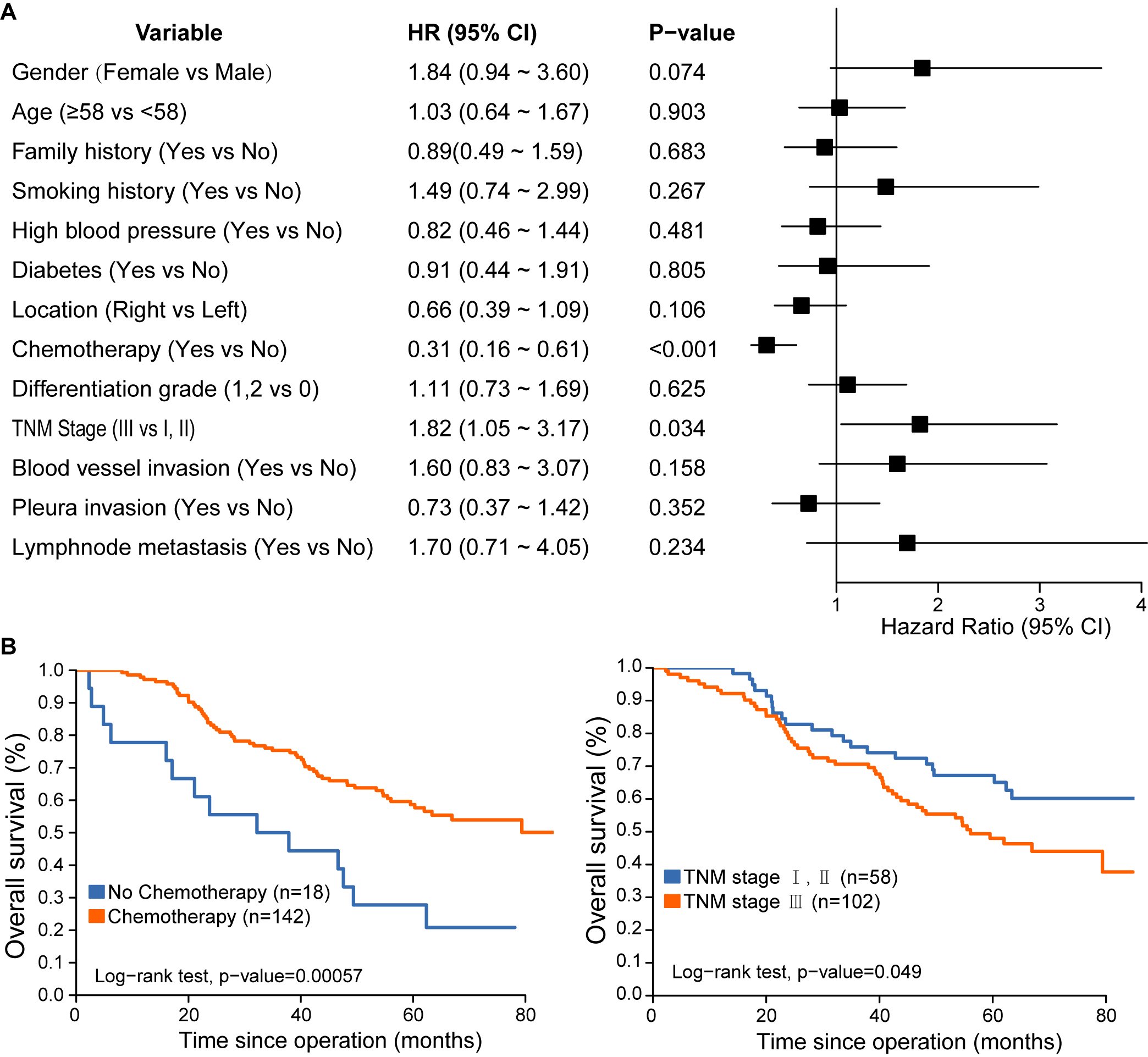

Supplement: Supplementary Figure 1 — Multivariate analysis of clinical information with prognosis. (A) The panel shows hazard ratios (HR) of clinicopathological characteristics for cancer-specific survival. (B) The Kaplan-Meier graphs depicting overall survival (OS) associated with chemotherapy and TNM stage. [file Image_1.TIF]

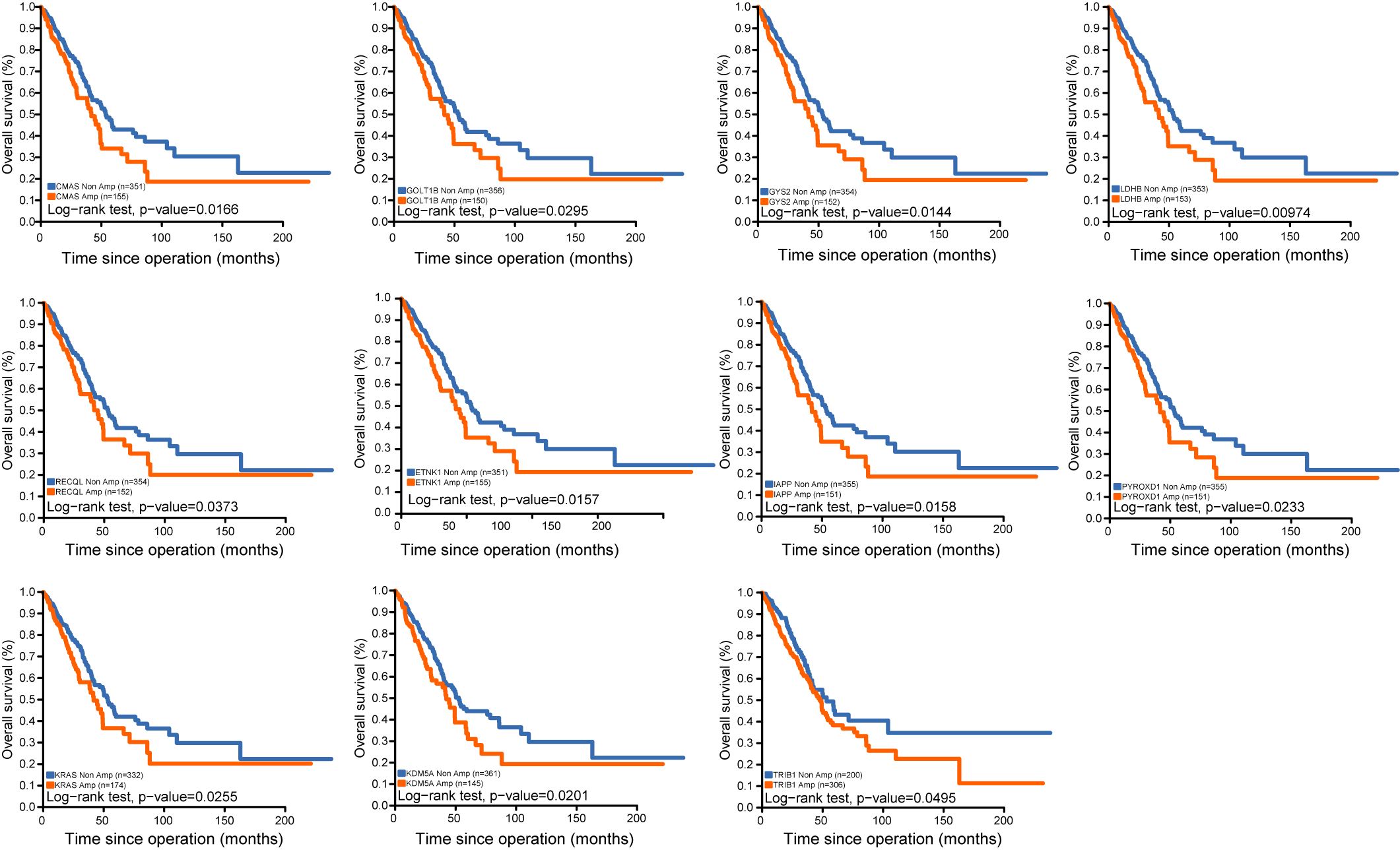

Supplement: Supplementary Figure 2 — Kaplan-Meier survival curves for survival of lung adenocarcinoma with CNA of select genes in TCGA dataset. [file Image_2.TIF]

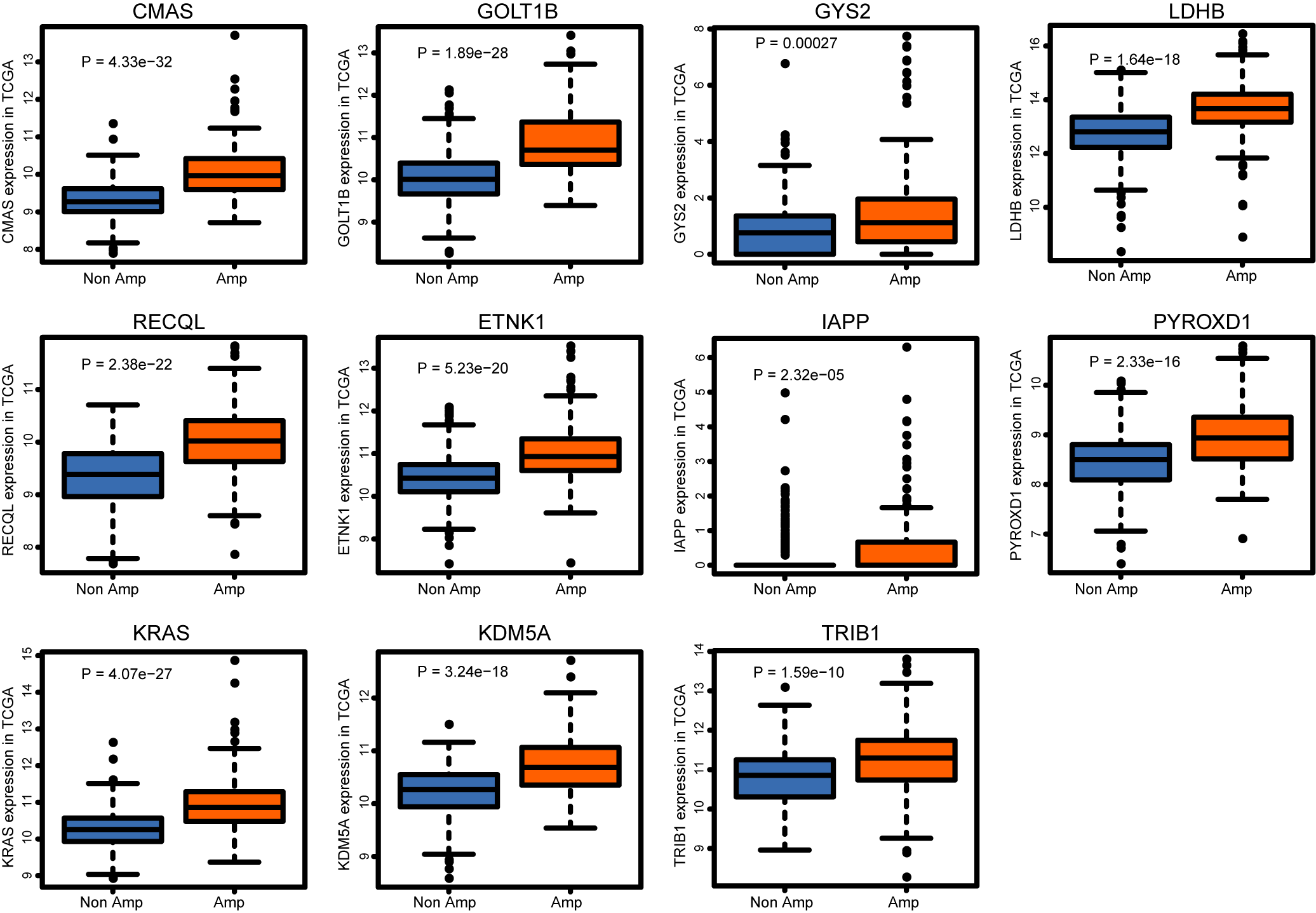

Supplement: Supplementary Figure 3 — Analyses of select genes expression levels by corresponding CNA status in 506 LUAD patients' tumors from the TCGA Project. [file Image_3.TIF]

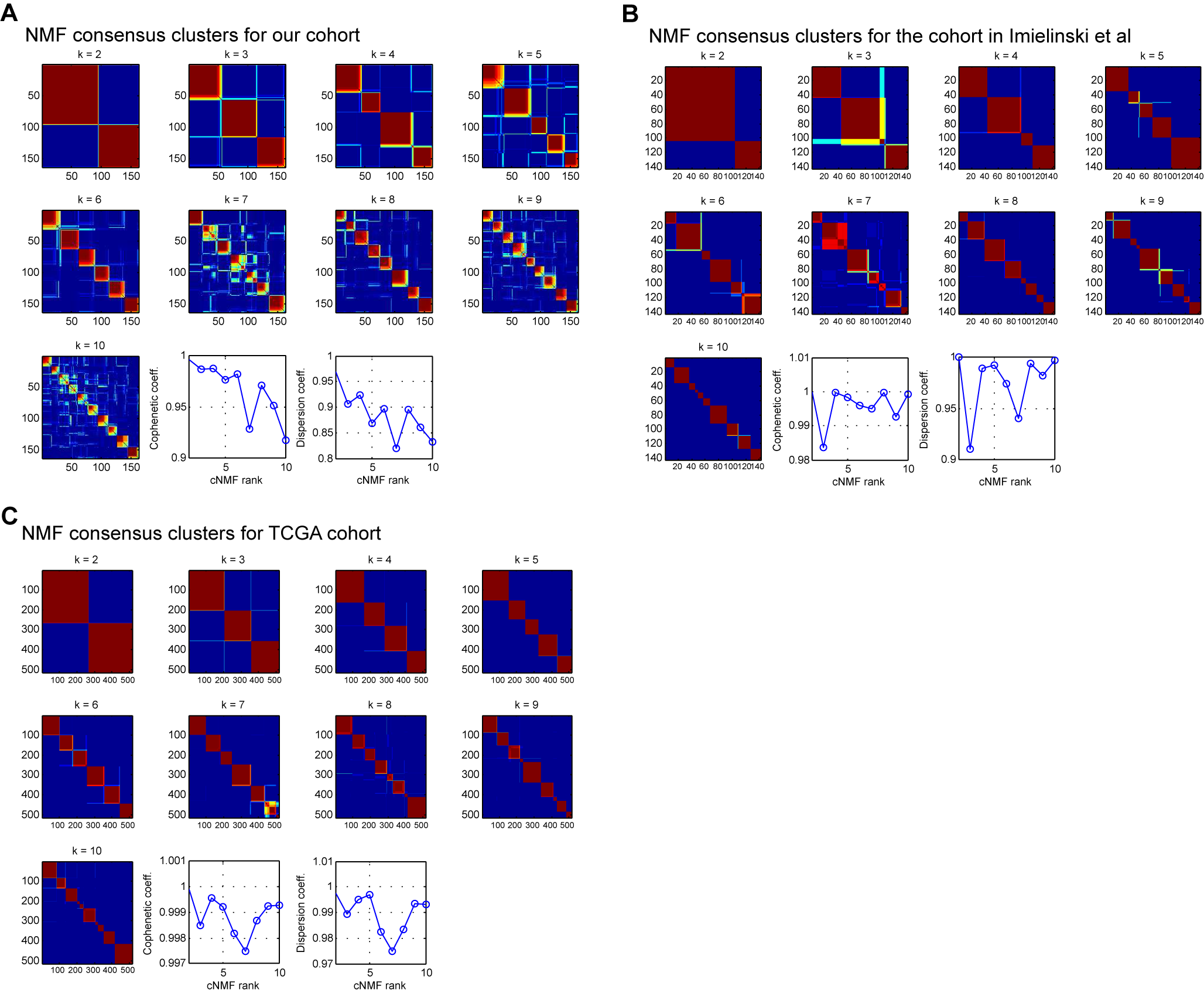

Supplement: Supplementary Figure 4 — NMF consensus clustering analysis and cophenetic coefficient for cluster k = 2 to k = 10. (A) our cohort. (B) The cohort from Imielinski et al. (C) TCGA cohort. [file Image_4.TIF]

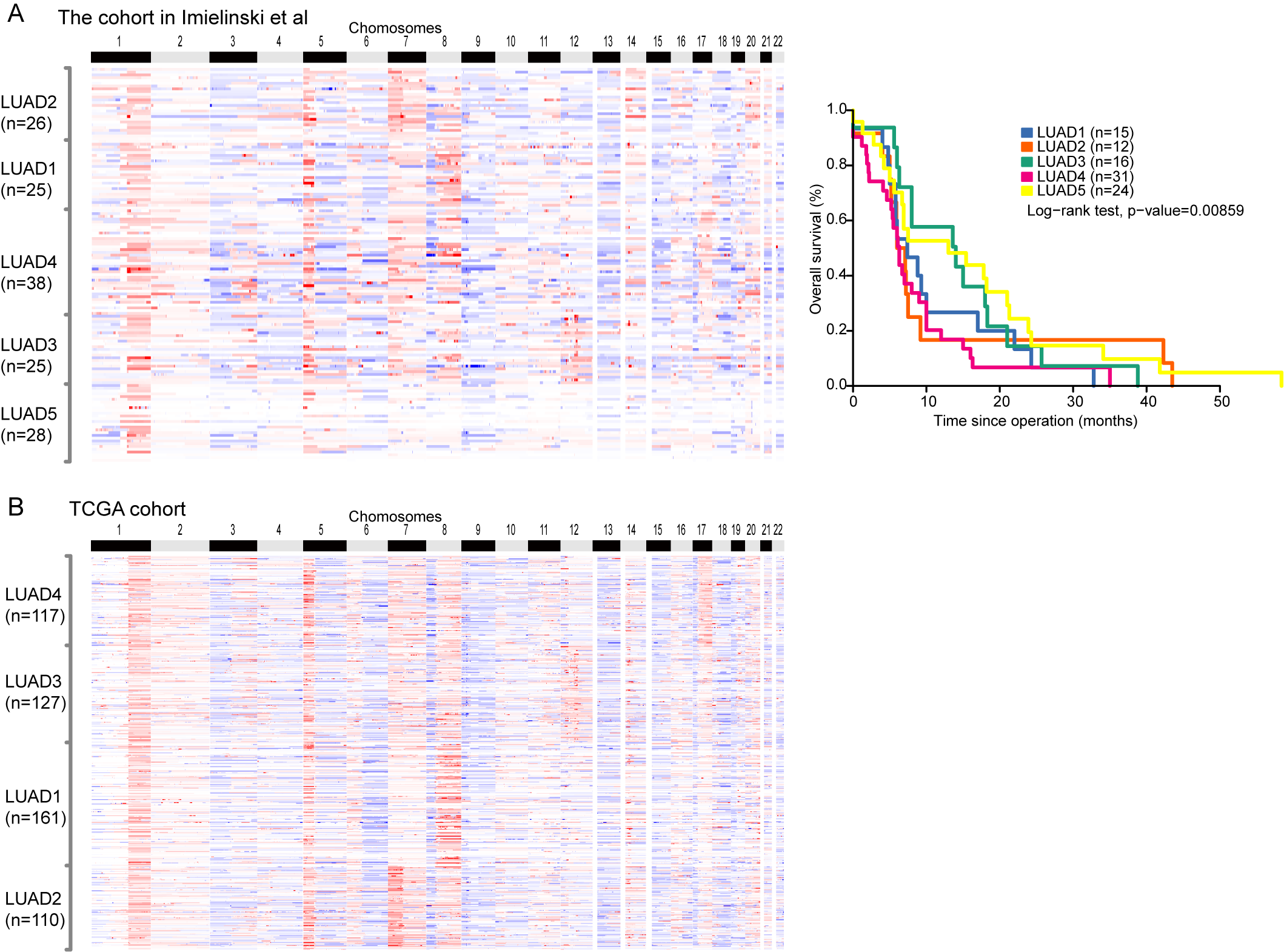

Supplement: Supplementary Figure 5 — Classification of validation datasets. (A) The cohort from Imielinski et al. (B) TCGA cohort. [file Image_5.TIF]

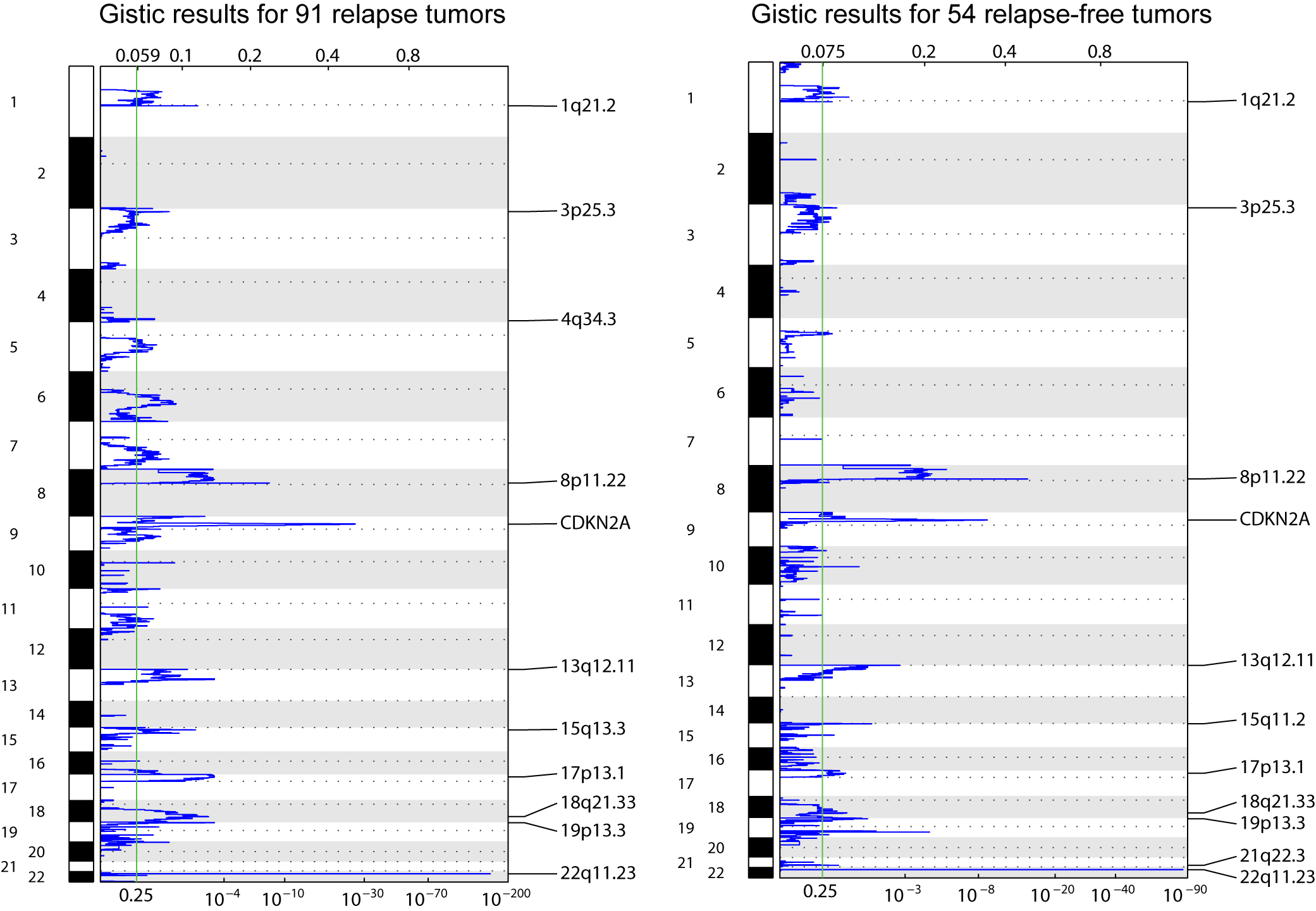

Supplement: Supplementary Figure 6 — GISTIC2.0 focal deletions (red) for post-operative relapse and relapse-free LUADs. Peaks with an FDR < 0.25 are annotated with candidate tumor suppressors or cytobands. [file Image_6.TIF]

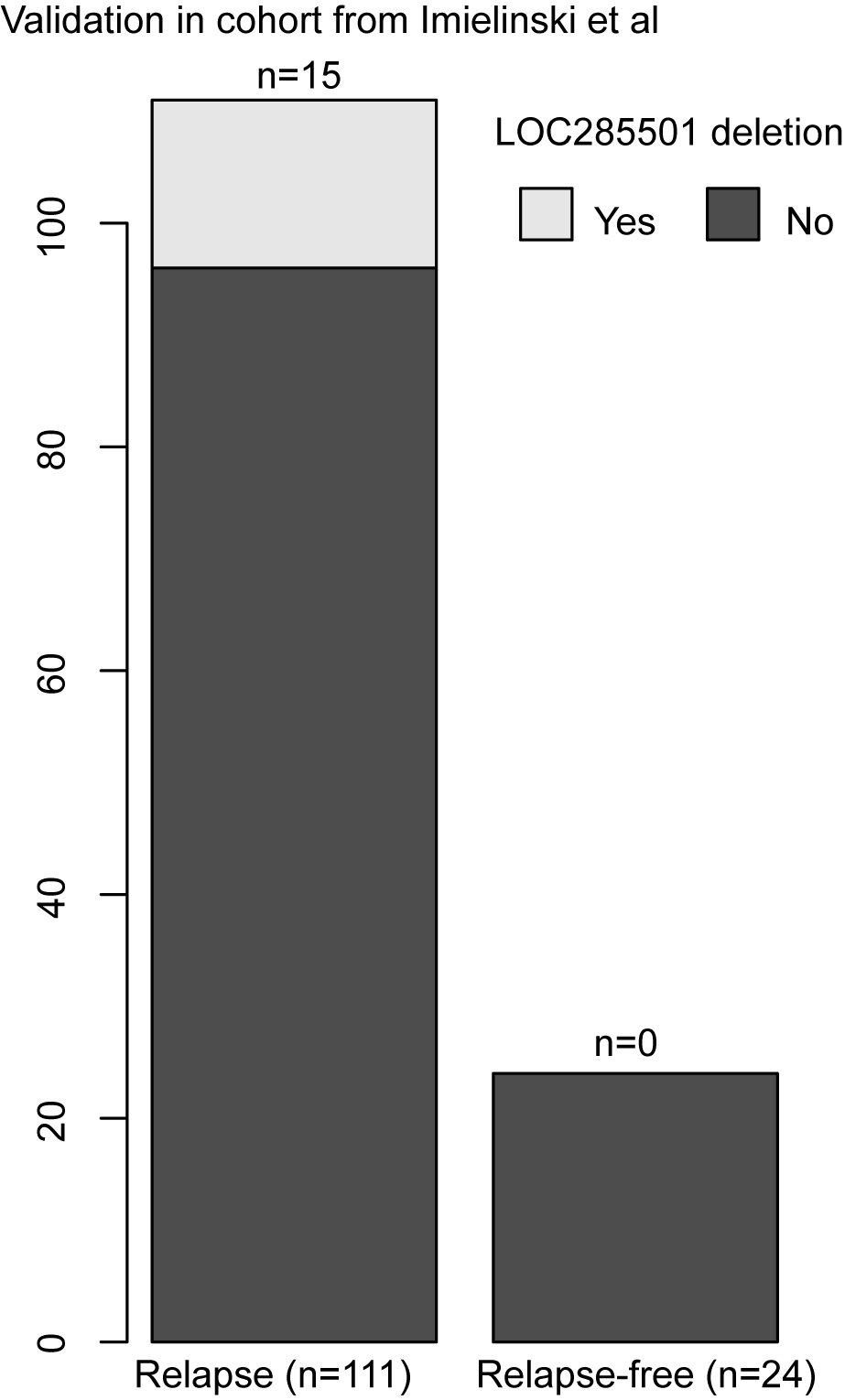

Supplement: Supplementary Figure 7 — Barplot shows the comparison on the LOC285501 deletion in validation sets. [file Image_7.TIF]
